# Fecal Microbiota Transplantation From Patients With Social Anxiety Disorder Is Associated With General Anxiety‐Like Behavior and Gut Microbiota Alterations in Mice

**DOI:** 10.1002/brb3.71561

**Published:** 2026-06-16

**Authors:** Yu‐Chao Feng, Xiao‐Xuan Liu, Huan Yu, Rui Liu, Ting‐Song Pang, Hua‐Ning Wang, Zheng‐Wu Peng

**Affiliations:** ^1^ Department of Psychiatry Xijing Hospital, Fourth Military Medical University Xi'an China; ^2^ Department of Shaanxi Key Laboratory for Animal Conservation Northwest University Xi'an China; ^3^ Department of Toxicology, Shaanxi Key Lab of Free Radical Biology and Medicine, The Ministry of Education Key Lab of Hazard Assessment and Control in Special Operational Environment, School of Public Health Fourth Military Medical University Xi'an China; ^4^ Department of College of Life Science Shaanxi Normal University Xi'an China

**Keywords:** fecal microbiota transplantation, general anxiety‐like behavior, gut microbiota, social anxiety disorder, tryptophan

## Abstract

**Purpose:**

Growing evidence implicates gut microbiota dysbiosis in social anxiety disorder (SAD), yet direct causal evidence remains limited. This study investigated whether fecal microbiota transplantation (FMT) from individuals with SAD was associated with general anxiety‐like behaviors and accompanying gut microbial and predicted metabolic alterations in mice.

**Methods:**

Fecal samples were collected from five patients diagnosed with SAD and five matched healthy controls and transplanted into antibiotic‐treated mice. Anxiety‐like behaviors were evaluated using the open‐field test (OFT) and elevated‐plus maze test (EPMT). Gut microbiota composition was assessed by 16S rRNA gene sequencing, microbial functional potential was inferred using PICRUSt2, and plasma tryptophan‐pathway metabolites were quantified.

**Results:**

Mice receiving SAD microbiota (SAD group) showed general anxiety‐like behaviors, characterized by more time spent in the periphery of the OFT and fewer entries and less time in the open arms of the EPMT compared to mice receiving healthy control microbiota (control group). Although α‐diversity did not differ significantly, β‐diversity was distinct between groups. The SAD group showed enrichment of Bacteroidota/Bacteroidales‐related bacteria (e.g., *Muribaculum*), whereas the control group had higher abundance of butyrate producers (e.g., *Butyricimonas*). Functional prediction indicated lower predicted abundance of selected DNA‐repair and biosynthetic pathways in the SAD group. Plasma tryptophan levels were nominally lower in the SAD group.

**Conclusions:**

These findings suggest that specific gut microbiota alterations and predicted functional pathway changes in individuals with SAD may be associated with anxiety‐related behavioral phenotypes, supporting the gut microbiome as a potential contributing factor to the pathophysiology of SAD.

## Graphical abstract

Fecal microbiota transplantation from patients with social anxiety disorder (SAD) into mice induces anxiety‐like behaviors, accompanied by gut microbiota alterations (increased Bacteroidota, decreased butyrate producers), downregulation of microbial metabolic pathways, and reduced plasma tryptophan, suggesting a causal role of gut dysbiosis in SAD‐related behaviors.

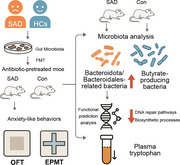

## 1 Introduction

Social anxiety disorder (SAD) is characterized by a persistent fear of being observed or judged in social settings. It affects about 7% of adults annually and roughly 12% over the lifespan (Hyett and McEvoy 2018). Symptoms often begin during adolescence and can persist for decades, impairing social, educational, and occupational functioning (Stein and Stein 2008). Although first‐line interventions—such as cognitive behavioral therapy, acceptance and commitment therapy, and selective serotonin reuptake inhibitors—provide relief for many patients, a substantial proportion either do not respond adequately or experience relapse (Kindred et al. 2022; Xian et al. 2024). These limitations highlight the need for alternative therapeutic strategies and a deeper understanding of the disorder's underlying pathophysiology.

The neurobiological basis of SAD is complex and multifactorial; there is growing recognition that the gastrointestinal microbiota play a key role in mental health through the gut–brain axis (GBA) (Margolis et al. 2021). This axis facilitates bidirectional communication between the gut and the brain via neural, immune, endocrine, and metabolic pathways (Mehta et al. 2025). Within the nervous system, intestinal microbes modulate central serotonergic, dopaminergic, and noradrenergic neurotransmission—systems long implicated in the pathophysiology of anxiety and depression. For instance, specific *Lactobacillus* and *Bifidobacterium* strains can increase serotonin levels in the hippocampus and frontal cortex of stressed rodents, whereas antibiotic‐induced microbiota depletion alters dopamine turnover and elevates striatal noradrenaline (Huang and Wu 2021). Gut bacteria produce neurotransmitter precursors and other neuromodulatory metabolites, stimulate vagal afferents, modulate the hypothalamic–pituitary–adrenal (HPA) axis, and influence systemic immune activity (Butler et al. 2019). On the immune side, toll‐like receptor 4 (TLR4) signaling activated by bacterial lipopolysaccharide (LPS) can trigger NF‐κB‐mediated inflammation, which has been linked to anxiety‐like behavior (Yang et al. 2019). Animals raised in germ‐free environments show exaggerated cortisol responses, reduced brain‐derived neurotrophic factor, and altered sociability, effects that can be normalized by colonization with specific microbes (Sudo et al. 2004). In rodent models, microbiota depletion reduces innate anxiety, whereas colonization with conventional microbiota or fecal microbiota transplantation (FMT) from anxious donors induced anxiety‐like behavior (Ren et al. 2025). Clinically, regulating intestinal microbiota can improve anxiety symptoms in roughly half of studies (Adıgüzel et al. 2025). Consequently, FMT is being explored as a therapeutic strategy to restore microbial ecosystems and modulate host behavior via microbiota‐GBA (Q. Zhang, Bi, et al. 2024).

Recent studies have increasingly uncovered the role of the gut microbiome in SAD. Shotgun sequencing of fecal samples from individuals with SAD revealed distinct differences in microbial community composition compared with healthy controls (Butler et al. 2023). FMT from SAD donors into antibiotic‐treated mice resulted in increased social avoidance and altered immune and oxytocin signaling pathways (Ritz et al. 2024). Across various anxiety models, specific microbial metabolites have been linked to behavioral changes. For instance, 4‐ethylphenyl sulfate, produced by certain gut bacteria, induces oligodendrocyte dysfunction and anxiety‐like behaviors in mice (Needham et al. 2022), while inhibiting succinate transport alleviates anxiety triggered by chronic infection (Luo et al. 2024). Despite these advances, the molecular pathways through which gut microbiota perturbations influence social behavior remain poorly understood.

In this study, we performed FMT using fecal microbiota from clinically diagnosed SAD patients and age‐matched healthy donors into antibiotic‐treated mice. Our primary objective was to determine whether SAD‐associated microbiota could induce anxiety‐like behaviors in recipient mice. Following transplantation, we assessed behavior using the open‐field test (OFT) and elevated plus maze test (EPMT). Additionally, we analyzed gut microbial diversity, predicted functional metabolic pathways, and measured plasma levels of tryptophan metabolites to explore potential mechanisms linking the gut microbiota to anxiety‐like behaviors.

## 2 Materials and Methods

### 2.1 Participants

The study was registered with the Chinese Clinical Trial Registry (registration number: ChiCTR2500097560) and approved by the Medical Ethics Committee of the First Affiliated Hospital of the Air Force Medical University (approval number: KY20242376‐X‐2). All procedures complied with the principles of the Declaration of Helsinki, and written informed consent was obtained from all participants prior to enrollment. In this case–control study, five patients diagnosed with SAD and five age‐ and sex‐matched healthy controls were recruited following structured clinical interviews. Participants were allocated into groups in a blinded manner by two psychiatrists. Stool samples were collected and stored at −80°C for subsequent use. Clinical and demographic characteristics of the participants are summarized in Table S1.

### 2.2 Animals and FMT

**FIGURE 1:**
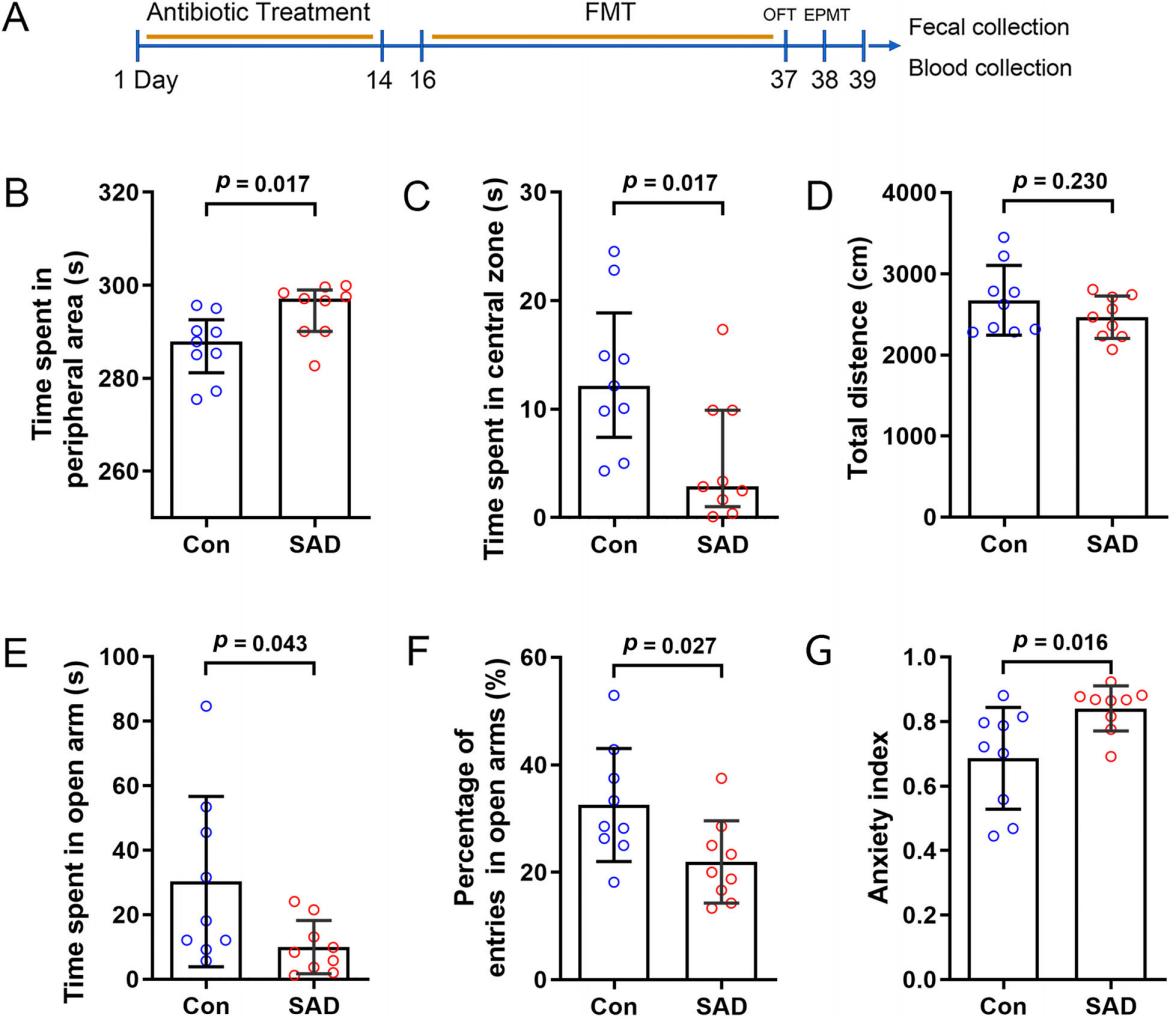
Transplantation of gut microbiota from SAD patients was associated with anxiety‐like behaviors in antibiotic‐treated mice: (A) animal experiment timeline; (B) time spent in peripheral area of OFT; (C) time spent in central zone of OFT; (D) total distance traveled in the OFT; (E) time spent in open arms of EPMT; (F) percentage of entries in open arms of EPMT; and (G) anxiety index in EPMT. Data in (B–C) are presented as median with interquartile range and were analyzed using the Mann–Whitney *U* test; data in (D–G) are presented as mean ± SD and were analyzed using independent‐samples *t*‐tests. Effect sizes (*r* or Cohen's *d*) and 95% confidence intervals are reported in the main text, *n* = 9 per group. Con, control group; EPMT, elevated‐plus maze test; FMT, fecal microbiota transplantation; OFT, open‐field test; SAD, social anxiety disorder group.

Two‐month‐old C57BL/6 male mice were obtained from the Animal Facility of the Air Force Medical University. The individual mouse was the experimental unit. After acclimatization, mice were randomly allocated to the control group or SAD group (*n* = 9 mice per group; total *n* = 18 recipient mice) using a random‐number procedure. The animals were housed in individually ventilated cages under standard conditions (22°C ± 2°C, 50% ± 4% humidity, 12‐h light/dark cycle) with free access to food and water. All experimental procedures were approved by the Animal Care and Utilization Committee of the Air Force Medical University and conducted in accordance with the Guidelines for the Care and Use of Experimental Animals and reported in compliance with the ARRIVE 2.0 guidelines (for details, see Table S2). FMT was performed according to established protocols (Zhang et al. 2022; Zhu et al. 2020). Mice were pretreated with a cocktail of metronidazole (0.5 g/L), amoxicillin (0.5 g/L), neomycin (0.5 g/L), and ampicillin (0.25 g/L) in their drinking water for 2 weeks. This antibiotic cocktail contains a nitroimidazole, two beta‐lactam penicillin agents, and an aminoglycoside and was used to broadly deplete susceptible anaerobic and aerobic bacteria before FMT. It should not be interpreted as producing a germ‐free state; residual bacteria and nonbacterial microorganisms, including fungi or protozoa, may persist or expand after treatment. Donor fecal material from SAD patients and healthy controls was collected, microbial suspensions were prepared and administered to the mice by oral gavage (10 µL/g body weight) for three consecutive weeks, starting 2 days after the final antibiotic treatment (Figure 1A). The recipient animals were designated as the SAD group and the control group.

### 2.3 Behavioral Assessments

All behavioral tests were conducted 24 h after the final FMT administration, under low‐light conditions, and video‐recorded using an automated tracking system (Ji Liang Co. Ltd., China). The apparatus was thoroughly cleaned with 70% ethanol between trials to eliminate olfactory cues. The experimenter was blinded to group allocation during testing and data acquisition. Behavioral outcomes were selected to quantify general anxiety‐like behavior and locomotor activity.

#### 2.3.1 Open‐Field Test

Each mouse was placed in the center of a square open‐field arena (50 × 50 cm^2^) and allowed to explore freely for 5 min. The total distance traveled (cm), time spent in the peripheral zone (defined as the area within 10 cm of the walls), and time spent in the central zone were analyzed. Total distance was used as an index of locomotor activity, whereas peripheral‐zone preference and reduced center‐zone time were interpreted as anxiety‐like behavior.

#### 2.3.2 Elevated Plus‐Maze Test

Mice were placed in the central zone of an elevated plus‐maze (35 cm long arms, 6 cm wide, elevated 50 cm above the floor), facing an open arm. Behavior was recorded for 5 min. Percentage of open‐arm entries, time spent in open arms, and anxiety index were analyzed. An arm entry was defined as all four paws entering the arm. The anxiety index was calculated as 1 − [(open‐arm time/total test time + open‐arm entries/total arm entries)/2]. Higher values indicate greater anxiety‐like behavior.

### 2.4 16S RRNA Gene Sequencing and Microbiome Analysis

Fecal samples were collected immediately after behavioral testing, snap‐frozen in liquid nitrogen, and stored at −80°C. Microbial genomic DNA was extracted from approximately 200 mg of feces using the HiPure Stool DNA Kit (Magen, China), following our previously established protocol (Y. H. Chen et al. 2023). The V3–V4 regions of the bacterial 16S rRNA gene were amplified in triplicate using primers 338F/806R on an ABI GeneAmp 9700 thermocycler. Pooled PCR products were quantified and sequenced. Raw FASTQ files underwent demultiplexing via custom Perl scripts and quality control using USEARCH v8.0. Operational taxonomic units (OTUs) were clustered at 97% similarity with UPARSE (v7.1) (Edgar 2013). Downstream analyses encompassed diversity metrics, principal coordinate analysis (PCoA), linear discriminant analysis (LDA), PICRUSt2 predictions, and correlation studies.

### 2.5 Quantification of Plasma Tryptophan Metabolites

Blood samples were centrifuged at 1600 rpm for 15 min to isolate plasma, which was then stored at −80°C. For metabolomic analysis, proteins were precipitated using precooled methanol/acetonitrile (1:1, v/v). The supernatant was lyophilized and reconstituted for analysis. Metabolite separation was performed on an Agilent 1290 Infinity UHPLC system with a maintained column temperature of 50°C. The mobile phase flow rate was 400 µL/min with an injection volume of 5 µL. The gradient elution program was as follows: 15% B (0–2 min), increased to 98% B (2–9 min), held at 98% B (9–11 min), returned to 15% B (11–11.5 min), and re‐equilibrated (11.5–14 min). Mass spectrometric detection was carried out using a 5500 QTRAP instrument in positive ion multiple reaction monitoring (MRM) mode. Ion source parameters were set as follows: temperature, 550°C; Gases 1 and 2, 55 psi; curtain gas, 40 psi; and ion spray voltage, +4500 V. Data were quantified using MultiQuant or Analyst software.

### 2.6 Statistical Analysis

The normality of data distribution was assessed using the Shapiro–Wilk test. Normally distributed continuous variables were compared using independent‐samples *t*‐tests, with effect sizes calculated as Cohen's *d* (mean difference divided by the pooled standard deviation) and 95% confidence intervals (CIs) calculated using the *t*‐distribution. Values of 0.20, 0.50, and 0.80 for Cohen's *d* represent small, medium, and large effects, respectively. Non‐normally distributed data were compared using the Mann–Whitney *U* test; effect sizes were calculated as *r* = |*Z*|/$\sqrt{N}$, where *Z* is the standard normal approximation of the Mann–Whitney *U* statistic with continuity correction, and *N* is the total sample size. Values of 0.10, 0.30, and 0.50 for *r* represent small, medium, and large effects, respectively. The Hodges–Lehmann estimator (median of all pairwise differences between groups) was used to estimate the median difference for nonparametric comparisons, with 95% CIs derived via bootstrap resampling (10,000 iterations). For microbiome data, differentially abundant taxa between groups were identified by LDA effect size (LEfSe) analysis, with an LDA score threshold of >2.5 and *p* < 0.05. KEGG pathway abundances were inferred from 16S rRNA gene sequencing data using PICRUSt2 and compared between groups, with Benjamini–Hochberg false discovery rate (FDR) correction applied to control for multiple comparisons. All statistical analyses were conducted using GraphPad Prism (v8.0), SPSS (v27.0), and the Majorbio cloud platform. A two‐sided *p* < 0.05 was considered statistically significant.

## 3 Results

### 3.1 FMT Is Associated With Anxiety‐Like Behaviors and Altered Gut Microbiota Diversity in Mice

In the OFT, mice receiving SAD microbiota displayed pronounced anxiety‐like behavior, characterized by a significantly increased proportion of time spent in the peripheral zone (Mann–Whitney *U* = 13.0, *Z* = −2.384, *p* = 0.017, *r* = 0.562, 95% CI: 0.177–0.843; Figure 1B) and significantly decreased time in the central zone (Mann–Whitney *U* = 68.0, *Z* = 2.384, *p* = 0.017, *r* = 0.562, 95% CI: 0.177–0.843; Figure 1C), compared to the control group. No significant difference in total distance traveled was detected between the SAD and control groups (2673.37 ± 428.79 vs. 2464.53 ± 261.27 cm; independent‐samples *t*‐test: *t*(16) = 1.248, *p* = 0.230, Cohen's *d* = 0.588, 95% CI: −0.435 to 1.612; Figure 1D), indicating that general locomotor activity was unaffected.

In the EPMT, SAD group mice spent significantly less time in the open arms (30.25 ± 26.40 vs. 9.97 ± 8.25 s; *t*(16) = 2.200, *p* = 0.043, Cohen's *d* = 1.037, 95% CI: −0.035 to 2.109; Figure 1E) and exhibited a significantly lower percentage of entries in open arms (32.54% ± 10.51% vs. 21.94% ± 7.68%; *t*(16) = 2.443, *p* = 0.027, Cohen's *d* = 1.152, 95% CI: 0.063–2.240; Figure 1F) compared to the control group. Consistently, the anxiety index was significantly higher in the SAD group than in the control group (0.84 ± 0.07 vs. 0.69 ± 0.16; *t*(16) = 2.682, *p* = 0.016, Cohen's *d* = 1.264, 95% CI: 0.158–2.370; Figure 1G). Taken together, these results indicated that FMT from SAD donors significantly increased general anxiety‐like behavior in recipient mice, without affecting general locomotor activity.

**FIGURE 2:**
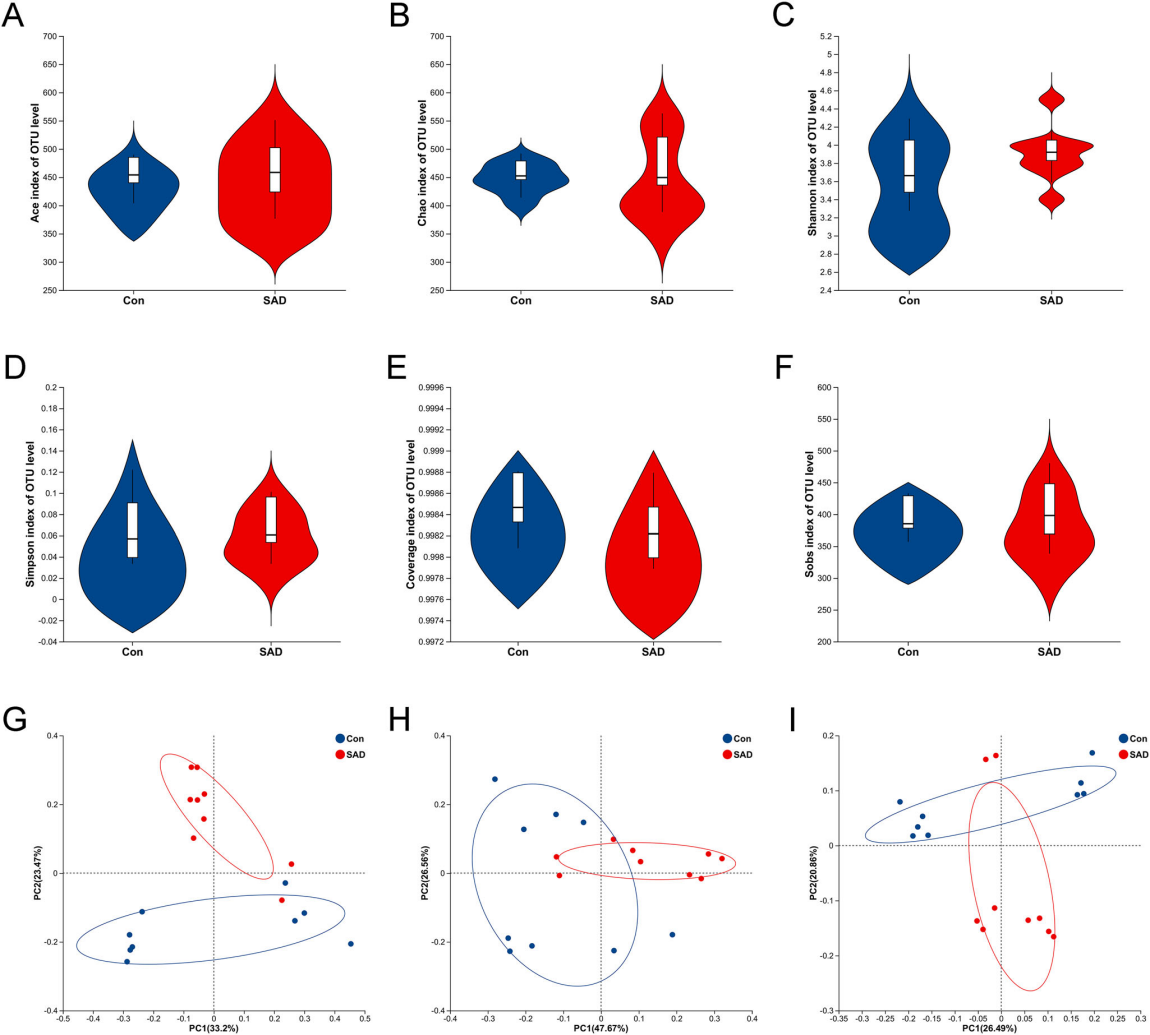
Comparison of alpha and beta diversity analyses between each group. (A–F) Alpha diversity indices: (A) Ace index, (B) Chao index, (C) Shannon index, (D) Simpson index, (E) Sobs index, and (F) coverage index. (G–I) Principal coordinate analysis (PCoA) based on (G) Bray–Curtis distance, (H) weighted UniFrac distance, and (I) unweighted UniFrac distance at the OTU level, demonstrating distinct separation between groups. The circle represents one value from individual mice. Con, control group; OUT, operational taxonomic unit; SAD, social anxiety disorder group.

**FIGURE 3:**
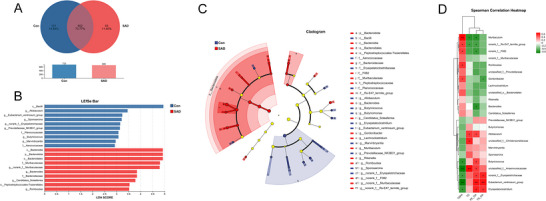
Altered microbial composition was observed in mice receiving gut microbiota from social anxiety disorder individuals: (A) Venn diagram and bar chart depicting the number of shared and unique operational taxonomic units (OTUs) between the control group (red) and social anxiety disorder group (blue); (B) linear discriminant analysis (LDA) effect size (LEfSe) bar plot showing microbial taxa with significant differential abundance in the control group (red) versus social anxiety disorder group (blue), where a higher LDA score indicates a greater contribution of the taxon's abundance to the group difference; only taxa with LDA scores >2.5 and an FDR‐corrected significance level of *p* < 0.05 are displayed; (C) cladogram illustrating bacterial taxa enriched in the control group (red) versus social anxiety disorder group (blue), with yellow dots representing taxa not significantly contributing to group differences; and (D) red and green squares denote positive and negative correlations, respectively, with color intensity proportional to the correlation magnitude (*p < 0.05; **p < 0.01). Con, control group; LDA, linear discriminant analysis; LEfSe, linear discriminant analysis effect size; SAD, social anxiety disorder group.

Analysis of α‐diversity indices (Ace, Chao, Shannon, Simpson, Sobs, and Coverage) revealed no significant intergroup differences (Figure 2A–F). Subsequent examination identified 723 OTUs at the species level in the control group, compared to 695 OTUs in the SAD group (Figure 3A). In contrast, β‐diversity analysis based on Bray–Curtis (*R* ^2^ = 0.1904, *p* = 0.001; Figure 2G), weighted UniFrac (*R* ^2^ = 0.2283, *p* = 0.007; Figure 2H), and unweighted UniFrac (*R* ^2^ = 0.155, *p* = 0.006; Figure 2I) metrics revealed clear clustering separation between the two groups. These results indicated that FMT from SAD donors was associated with shifts in beta diversity, but not alpha diversity, of the recipient gut microbiota.

### 3.2 FMT Alters Microbial Composition and Functional Potential

Distinct taxonomic profiles were observed between the control and SAD groups, as confirmed by LEfSe analysis (Figure 3B,C). The control group was enriched in members of the class Bacilli; families Planococcaceae, Aerococcaceae, and Erysipelatoclostridiaceae; and genera including *Eubacterium_ventriosum_group*, *Allobaculum*, *Sporosarcina*, *norank_f_Erysipelotrichaceae*, *Prevotellaceae_NK3B1_group*, *Butyricimonas*, *Marvinbryantia*, *Butyricicoccus*, and *Erysipelatoclostridium*. In contrast, the SAD group showed enrichment in the phylum Bacteroidota; class Bacteroidia; orders Bacteroidales and Peptostreptococcales‐Tissierellales; family Muribaculaceae, Bacteroidaceae, F082, Peptostreptococcaceae, and Rs‐E47_termite_group; and genera such as *Bacteroides*, *norank_f_Muribaculaceae*, *Candidatus_Soleaferrea*, *Romboutsia*, *Gordonibacter*, *Lachnoclostridium*, *Muribaculum*, *Rikenella*, *norank_f_F082*, and *norank_f_Rs‐E47_termite_group*.

Correlation analysis at the genus level revealed several significant associations between gut microbiota abundance and anxiety‐like behavioral measures. Time spent in the peripheral zone correlated positively with *Muribaculum*, *norank_f_Rs‐E47_termite_group*, *norank_f_F082*, *Romboutsia*, and *Gordonibacter*, and negatively with *Butyricimonas*, *unclassified_f_Anaerovoracaceae*, *norank_f_Erysipelotrichacea*, *Eubacterium_ventriosum_group*, and *Erysipelatoclostridium*. Total distance traveled correlated negatively with *Muribaculum* and *norank_f_Rs‐E47_termite_group*, and positively with *Allobaculum*, *unclassified_f_Christensenellaceae*, and *unclassified_f_Anaerovoracaceae*. Percentage of open‐arm entries correlated negatively with *Muribaculum*, *norank_f_Rs‐E47_termite_group*, *norank_f_F082*, and *Bacteroides* and positively with *Allobaculum*, *Butyricimonas*, *unclassified_f_Anaerovoracaceae*, *norank_f_Erysipelotrichaceae*, *Eubacterium_ventriosum_group*, and *Erysipelatoclostridium*. Time spent in the open arms correlated negatively with *Gordonibacter* and positively with *norank_f_Erysipelotrichaceae*, *Eubacterium_ventriosum_group*, and *Erysipelatoclostridium* (Figure 3D).

**FIGURE 4:**
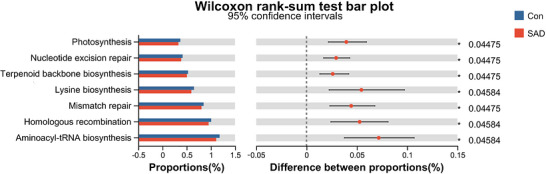
Differences in key predicted metabolic pathways of the gut microbiota between Con and social anxiety disorder. The left panel shows the mean abundance of each differentially predicted metabolic pathway in the two groups. The middle panel displays the difference in mean abundance with 95% confidence intervals. The right panel indicates the FDR‐corrected *p* value for each pathway. Con, control group; SAD, social anxiety disorder group.

Using PICRUSt2 to infer functional potential from 16S rRNA gene sequencing data, six KEGG pathways showed significant differences between groups after FDR correction. Predicted DNA‐repair pathways, including mismatch repair (*p* = 0.046), homologous recombination (*p* = 0.046), and nucleotide excision repair (*p* = 0.045), were lower in the SAD group. Predicted metabolic pathways also showed lower abundance, including terpenoid backbone biosynthesis (*p* = 0.045), lysine biosynthesis (*p* = 0.046), and aminoacyl‐tRNA biosynthesis (*p* = 0.046) (Figure 4).

### 3.3 FMT Influences Plasma Tryptophan Metabolite Levels

We quantified 20 tryptophan pathway metabolites in two groups (Table 1). Tryptophan showed the largest nominal group difference, with lower levels in the SAD group (6652.81 ± 1092.38 ng/mL) compared to the control group (8584.43 ± 1678.80 ng/mL; *t*(16) = 2.89, nominal *p* = 0.01, Cohen's *d* = 1.36). However, this difference did not survive Benjamini–Hochberg FDR correction (*q* = 0.21). None of the other 19 metabolites, including serotonin and kynurenine derivatives, showed significant differences at either the nominal or FDR‐corrected level. These exploratory findings suggest that SAD‐associated microbiota may alter host tryptophan availability, though this requires validation by targeted metabolomics.

**TABLE 1:** Concentrations of plasma tryptophan metabolites in each group (ng/mL).

Compounds	Con (*n* = 9)	SAD (*n* = 9)	*t*/*U*	*p*	*P*‐FDR	Effect size
l‐kynurenine	244.43 ± 113.50	173.29 ± 41.62	1.77	0.10	0.38	*d* = 0.83
Picolinic acid	47.67 (36.07, 54.46)	43.85 (34.66, 74.60)	42.00	0.93	0.93	*r* = 0.02
Tryptamine	0.42 ± 0.12	0.51 ± 0.19	1.22	0.24	0.53	*d* = 0.57
5‐Hydroxyindole‐3‐acetic acid	45.13 ± 9.44	44.72 ± 7.80	0.10	0.92	0.93	*d* = 0.05
3‐Indoxyl sulfate	1550.78 ± 1200.43	962.14 ± 464.90	1.37	0.19	0.53	*d* = 0.65
3‐Hydroxyl‐l‐kynurenine	10.29 (6.97, 18.28)	11.92 (5.77, 13.67)	45.00	0.72	0.93	*r* = 0.08
Xanthurenate acid	30.39 (25.56, 38.61)	30.67 (23.47, 37.71)	44.00	0.80	0.93	*r* = 0.06
Cinnavalininate acid	190.60 ± 89.98	234.24 ± 121.70	0.87	0.40	0.73	*d* = 0.41
5‐Hydroxy‐l‐tryptophan	22.82 ± 16.51	36.39 ± 22.38	1.47	0.16	0.53	*d* = 0.69
Quinolinic acid	89.80 (86.37, 106.40)	97.81 (93.08, 126.30)	29.00	0.33	0.66	*r* = 0.25
*N*‐formyl‐kynurenine	21.01 ± 5.68	16.79 ± 3.03	1.97	0.07	0.38	*d* = 0.93
Indole‐3‐lactic acid	247.77 ± 36.27	250.20 ± 75.09	0.09	0.93	0.93	*d* = 0.04
Indole‐3‐acetaldehyde	231.80 ± 34.23	213.40 ± 28.44	1.24	0.23	0.53	*d* = 0.59
Kynurenate	0.50 ± 0.14	0.46 ± 0.10	0.62	0.55	0.84	*d* = 0.29
Indoxyl‐β‐d‐glucuronide	95.91 ± 72.76	49.00 ± 26.64	1.82	0.09	0.38	*d* = 0.86
Indole‐3‐propionic acid	429.60 (282.40, 521.20)	322.60 (217.00, 564.10)	47.00	0.60	0.85	*r* = 0.13
Serotonin	488.05 ± 460.18	367.38 ± 226.47	0.71	0.49	0.82	*d* = 0.33
Indoleacetate	106.16 ± 25.81	108.71 ± 42.79	0.15	0.88	0.93	*d* = 0.07
Indole‐3‐carboxaldehyde	22.84 ± 10.18	14.50 ± 7.70	1.96	0.07	0.38	*d* = 0.92
Tryptophan	8584.43 ± 1678.80	6652.81 ± 1092.38	2.89	0.01	0.21	*d* = 1.36

*Note*: Data are presented as mean ± SD (normally distributed) or median (25th, 75th percentile) (non‐normally distributed). Statistical comparisons and effect sizes are described in the Methods. *P*‐FDR, false discovery rate‐corrected *q* value (Benjamini–Hochberg method). No metabolite remained significant after FDR correction (all *q* > 0.05).

## 4 Discussion

In this study, we found that FMT from individuals with SAD was associated with general anxiety‐like behaviors in antibiotic‐treated mice. Recipients of SAD microbiota displayed clear behavioral changes, including increased time spent in the periphery of the OFT and avoidance of the open arms in the EPMT, consistent with an anxiety‐like phenotype. Although no significant differences in α‐diversity were observed between groups, we identified pronounced shifts in microbial community structure (β‐diversity), accompanied by specific taxonomic alterations—such as increased relative abundance of *Bacteroides*, *Muribaculum*, and *Romboutsia* and decreased levels of butyrate‐producing genera, including *Butyricimonas* and the *Eubacterium_ventriosum_group*. Functional prediction analyses further indicated a downregulation in the SAD group of microbial pathways involved in DNA repair, homologous recombination, and amino acid biosynthesis. In line with these functional predictions, we observed nominally lower plasma tryptophan levels in SAD group. Our findings are consistent with a growing body of evidence suggesting that the gut microbiome contributes to the regulation of anxiety‐related behavior. Previous studies have shown that germ‐free and antibiotic‐treated rodents exhibit heightened HPA axis responses and increased anxiety, which can be ameliorated by microbial colonization (Leclercq et al. 2017). Similarly, chronic *Toxoplasma gondii* infection has been reported to promote anxiety‐like behavior through microbiota‐dependent mechanisms, an effect reversible with antibiotic treatment (Luo et al. 2024). Furthermore, FMT from patients with generalized anxiety disorder (GAD), irritable bowel syndrome (IBS), or anorexia nervosa has been shown to reduce exploratory behavior and increase anxiety in recipient mice (S. Chen et al. 2024; De Palma et al. 2017; Hata et al. 2019). A recent study also reported that FMT from SAD patients enhanced social fear in mice (Ritz et al. 2024). In agreement with these reports, our FMT experiments are consistent with the interpretation that microbiota from SAD is associated with increased anxiety‐like behavior in standardized behavioral tests.

Notably, although certain psychiatric conditions are characterized by reduced microbial diversity, our study found no significant differences in α‐diversity, consistent with other SAD and GAD reports, but identified clear restructuring of β‐diversity. This pattern underscores that specific microbial consortia, rather than overall richness, are likely critical in shaping behavioral outcomes. In our study, the control group showed enrichment of *Bacilli*, *Planococcaceae*, *Aerococcaceae*, and multiple butyrate‐producing genera, including *Butyricimonas*, *Butyricicoccus*, *Eubacterium_ventriosum_group*, and *Allobaculum, and these microbes were positively correlated with time spent in the open arms* (Scaldaferri et al. 2023). This coincided with earlier evidence that dysbiosis in anxiety and depression is featured by decreased butyrate‐producing microbes such as *Lachnospira*, *Faecalibacterium*, and *Butyricimonas*, accompanied by elevated pro‐inflammatory microbes (Maltz et al. 2019). Butyrate facilitates energy metabolism of colonic epithelial cells, maintains intestinal barrier integrity, and suppresses inflammatory signaling (Korsten et al. 2023). Accumulating evidence confirms the anxiolytic effects of these microbes. Butyrate‐producing taxa like *Butyricimonas* are more prevalent in healthy individuals, and they exert antianxiety effects by upregulating hippocampal BDNF and activating AMPK as well as PI3K‐Akt signaling pathways (S. Chen et al. 2024). However, the present study identified a positive correlation between the abundance of *Allobaculum* and both total distance traveled and open‐arm entries. Such discrepancies may reflect functional heterogeneity within the genus. Differences in host genetic background, diet, and microbial community context may further contribute to these divergent outcomes (San Gabriel et al. 2024). *Erysipelotrichaceae* and *Erysipelatoclostridium* were also enriched in control mice and correlated with reduced anxiety‐like behavior. However, other studies have linked this family to fear and stress, suggesting considerable functional diversity within the family (Carlson et al. 2021; J. Li, Fan, et al. 2024). Similarly, *Planococcaceae* and *Aerococcaceae*, both enriched in our control group, also present complex and context‐dependent associations that warrant further investigation (Wang et al. 2024; N. Zhang, Gao, et al. 2024; Zheng et al. 2019).

The SAD group exhibited marked enrichment in Bacteroidota/Bacteroidales‐related bacteria, including *Bacteroides*, *Muribaculaceae* (e.g., *Muribaculum*, *norank_f_Muribaculaceae*), *Peptostreptococcaceae*, and the unclassified lineages *F082* and Rs‐E47_termite_group. Many of these taxa carry endotoxins such as LPS or generate amino acid‐derived metabolites that can trigger inflammation and HPA axis activation (Zhou et al. 2022). Ritz et al. (2024) reported elevated *Bacteroides* species in SAD group, supporting their potential pro‐inflammatory and anxiety‐promoting roles. Other genera enriched in SAD include *Rikenella* and *Lachnoclostridium*. This contrasts with chronic sleep deprivation models, where *Rikenella* depletion was linked to mood impairment (L. Li, Meng, et al. 2024). In our SAD cohort, the increased abundance of *Rikenella* may suggest that, within a dysbiotic environment, certain *Rikenella* species could indirectly sustain inflammation by cross‐feeding pro‐inflammatory bacteria (Fernández‐Calleja et al. 2018). The role of *Lachnoclostridium* remains unclear; however, its involvement in choline metabolism and acetate production indicates a potential capacity to modulate neurotransmitter systems (Cai et al. 2022), which warrants further exploration.

*Romboutsia*, which is enriched in the SAD group and positively correlated with anxiety, shows a different pattern from its abundance in healthy controls in a psychological stress model (Geng et al. 2019), further emphasizing the context‐dependency of microbial function. *Gordonibacter* predominated in mice manifesting SAD and is inversely associated with open‐arm duration. The aromatic metabolites it generates are able to interfere with serotonergic signaling pathways, offering a potential mechanistic basis for its anxiety‐linked traits (Butler et al. 2023). *Peptostreptococcaceae* and *Peptostreptococcales–Tissierellales* were also enriched in SAD mice. These clostridial taxa are often associated with toxic metabolite production or fecal odor compounds, which may compromise intestinal barrier integrity and influence mood via vagal afferent signaling (Siopi et al. 2023; Takajo et al. 2019). The unclassified lineages *F082* and *Rs‐E47_termite_group*, likewise enriched in SAD and anxiety‐correlated, are poorly studied members of the Bacteroidales order. They are hypothesized to produce LPS similar to *Bacteroides*, though this requires experimental validation (d'Hennezel et al. 2017).

PICRUSt2 functional prediction indicated that pathways involving mismatch repair, homologous recombination, lysine biosynthesis, and aminoacyl‐tRNA biosynthesis were significantly downregulated in the SAD group. These predicted functional alterations suggest that SAD‐associated microbiota may establish a metabolic milieu that heightens host anxiety susceptibility by impairing DNA repair and limiting amino acid availability (Arat Celik et al. 2024; Raza et al. 2016; Smriga and Torii 2003; Pu et al. 2021). However, it is important to acknowledge that PICRUSt2 predictions are inferred from 16S rRNA gene sequence data and may not fully capture the functional diversity of the microbiome. These predictions require validation through metagenomic or metabolomic approaches.

Tryptophan metabolism proceeds through three principal pathways: the serotonin pathway, the kynurenine pathway, and the indole pathway. To experimentally validate these functional predictions, we analyzed plasma tryptophan metabolites and found that tryptophan levels were nominally lower in the SAD group, but this difference did not survive correction for multiple comparisons. None of the other measured metabolites showed significant differences between groups. Taken together, these results indicated that, under the present experimental conditions, FMT from SAD donors did not produce robust alterations in the measured tryptophan metabolites, which may be due to limited sample size.

This study has several limitations that should be considered when interpreting the results. First, the small sample size of human donors limits the statistical power and generalizability of the findings. The behavioral differences observed, while consistent, were modest in magnitude, and a larger donor cohort is needed to confirm the reliability of the SAD‐associated microbial profile identified. Second, although antibiotic pretreatment effectively depleted endogenous microbiota, residual fungal or archaeal communities may have persisted and potentially interacted with transplanted bacteria, confounding the observed effects. Third, the open field test and elevated plus maze are widely used assays for anxiety‐like behavior in rodents; however, they primarily measure exploratory conflict and general anxiety rather than social anxiety. Fourth, all recipients were male, limiting the generalizability of the findings to females, given established sex differences in the prevalence and manifestation of anxiety disorders. Additionally, we did not perform longitudinal microbiota profiling of donor samples, recipient baseline samples after antibiotic depletion, and post‐FMT samples at multiple time points, nor did we provide strain‐level evidence of donor‐to‐recipient microbial engraftment. Such analyses would strengthen the evidence that the observed behavioral and microbial changes are specifically attributable to the transplanted SAD‐associated microbiota. Future studies incorporating larger and sex‐balanced donor cohorts, metagenomic sequencing, social interaction paradigms, and multi‐omic approaches are warranted to validate and extend these findings.

## 5 Conclusion

In conclusion, this study adds to the microbiota‐GBA literature by examining whether gut microbiota from individuals with SAD are linked to anxiety‐related behavioral patterns in a preclinical model. The observation of both reduced butyrate‐producing taxa and increased pro‐inflammatory bacteria in the SAD microbiome raises the possibility that microbial mediators, such as short‐chain fatty acid signaling and immune‐related pathways, may contribute to anxiety vulnerability. Moving forward, combining longitudinal sampling, metagenomics, and behaviorally specific paradigms could help clarify these microbial contributions and explore whether microbiome‐targeted approaches might offer adjunctive therapeutic potential for SAD.

## Author Contributions

**Huan Yu**: funding acquisition, visualization, data curation. **Xiao‐Xuan Liu**: investigation, formal analysis, writing – original draft. **Ting‐Song Pang**: validation, investigation. **Yu‐Chao Feng**: writing – original draft, investigation. **Zheng‐Wu Peng**: conceptualization, funding acquisition, writing – review and editing, supervision, project administration. **Hua‐Ning Wang**: writing – review and editing, supervision. **Rui Liu**: funding acquisition, methodology.

## Funding

This work was funded by the National Natural Science Foundation of China (82171512) and the Boost plan of Xijing Hospital (No. XJZT25QN50 and LHJJ24YF06).

## Conflicts of Interest

The authors declare no conflicts of interest.

## Supporting information



Supplementary Table: brb371561‐sup‐0001‐TableS1.xlsx

Supplementary Table: brb371561‐sup‐0002‐TableS2.docx

## Data Availability

The data will be made available on request.
