# Supplementary material for: Fecal Microbiota Transplantation From Patients With Social Anxiety Disorder Is Associated With General Anxiety‐Like Behavior and Gut Microbiota Alterations in Mice
Source: Brain Behav. 2026 Jun 16;16(6):e71561. doi: 10.1002/brb3.71561 (PMC13272884; doi:10.1002/brb3.71561)
Supplement: Supplementary file 2 — Supplementary Table: brb371561‐sup‐0002‐TableS2.docx [file BRB3-16-e71561-s002.docx]

**ARRIVE Guidelines 2.0 Completed Checklist**

*Animal Research: Reporting of In Vivo Experiments*

| **Manuscript title** | Fecal Microbiota Transplantation from Patients with Social Anxiety Disorder Is Associated with General Anxiety-like Behavior and Gut Microbiota Alterations in Mice |
| --- | --- |
| **Corresponding authors** | Hua-Ning Wang and Zheng-Wu Peng |

**The ARRIVE Essential 10**

| **ARRIVE item** | **Requirement from ARRIVE 2.0** | **Location in revised manuscript** | **Information reported / note for compliance** | **Status** |
| --- | --- | --- | --- | --- |
| **1. Study design** | For each experiment, provide brief details of the study design, including the groups being compared, control groups, and the experimental unit. | Abstract; Sections 2.1-2.5; Figure 1A | The study compares recipient mice given fecal microbiota from social anxiety disorder donors with recipient mice given fecal microbiota from healthy controls. The individual mouse is stated as the experimental unit. Donor groups included five social anxiety disorder patients and five matched healthy controls. Recipient mice were assigned to the control group or social anxiety disorder group before behavioral, microbiome, functional-prediction and plasma tryptophan analyses. | **Reported** |
| **2. Sample size** | Specify the exact number of experimental units allocated to each group and the total number in each experiment; explain how sample size was decided and report any a priori calculation if performed. | Sections 2.1 and 2.2; Results; figure captions | The revised manuscript reports five donors per human group and C57BL/6 male recipient mice allocated to two groups (n = 9 mice per group; total n = 18 recipient mice). The results and figure captions should consistently report the exact n for each behavioral, microbiome and metabolite analysis. No formal a priori sample-size calculation is described; this should be stated transparently as a limitation or as a feasibility-based exploratory design. | **Partially reported** |
| **3. Inclusion and exclusion criteria** | Describe criteria for including and excluding animals or data points, state whether criteria were set a priori, report any exclusions, and report exact n for each analysis. | Section 2.1; Section 2.6; Results and figure captions | Human participants were diagnosed with social anxiety disorder or selected as matched healthy controls following structured clinical interviews. The animal exclusion criteria and data-point exclusion rules are not fully described in the text. If no animal or data-point exclusions occurred, add an explicit statement: "No animals, experimental units, or data points were excluded from the analyses." Exact n values should be retained in every figure caption and statistical result. | **Partially reported** |
| **4. Randomisation** | State whether randomisation was used to allocate experimental units to groups; provide the method used to generate the sequence; describe methods used to minimise potential confounders. | Sections 2.1 and 2.2 | Recipient mice were randomly allocated to the control group or social anxiety disorder group using a random-number procedure after acclimatisation. Potential procedural confounders were partly controlled by standardised housing, identical behavioral test duration, low-light testing, and ethanol cleaning between trials. Cage-position allocation, order of testing and order of sample processing should be stated if available. | **Partially reported** |
| **5. Blinding** | Describe who was aware of group allocation during allocation, experiment conduct, outcome assessment and data analysis. | Sections 2.1 and 2.3 | The manuscript states that participant grouping was handled by two psychiatrists and that the experimenter was blinded to group allocation during behavioral testing and data acquisition. The blinding status during fecal suspension preparation, animal allocation and statistical analysis should be stated if available. | **Partially reported** |
| **6. Outcome measures** | Clearly define all outcome measures; for hypothesis-testing studies, specify the primary outcome used for sample-size determination. | Sections 2.3-2.5; Section 2.6; Results 3.1-3.3 | Behavioral outcomes include OFT total distance, peripheral-zone time, central-zone time, and EPMT open-arm measures. Biological outcomes include fecal 16S rRNA sequencing-derived diversity/taxonomic profiles, PICRUSt2-predicted functional potential, and plasma tryptophan-pathway metabolites. The primary outcome is general anxiety-like behavior assessed by OFT and EPMT. The manuscript should clarify that no outcome was used for formal sample-size calculation if no power analysis was performed. | **Partially reported** |
| **7. Statistical methods** | Provide details of statistical methods for each analysis, including software; describe assumption checks and actions if assumptions were not met. | Section 2.6; Results; figure captions | Normality was assessed with the Shapiro-Wilk test. Parametric data were compared by independent-samples t-tests with Cohen's d and 95% confidence intervals. Non-parametric data were compared by Mann-Whitney U tests with r effect sizes and Hodges-Lehmann estimates. Microbiome analyses included LEfSe, PICRUSt2 and Benjamini-Hochberg FDR correction for multiple comparisons. Software included GraphPad Prism, SPSS and the microbiome cloud platform. | **Reported** |
| **8. Experimental animals** | Provide species-appropriate details, including species, strain/substrain, sex, age/developmental stage, weight if relevant, provenance, health/immune status, genetic status and previous procedures. | Section 2.2 | The manuscript reports two-month-old male C57BL/6 mice obtained from the Animal Facility of the Air Force Medical University. The source, strain, sex and age are present. Body weight range, substrain if applicable, health status, immune status, genetic modification status and previous procedures are not fully described and should be added if available. | **Partially reported** |
| **9. Experimental procedures** | For each group, describe what was done, how, when/how often, where including acclimatisation, and why the procedure was performed. | Sections 2.2-2.5; Figure 1A | The manuscript describes antibiotic pretreatment with metronidazole, amoxicillin, neomycin and ampicillin for two weeks; FMT by oral gavage at 10 uL/g body weight for three consecutive weeks starting two days after antibiotics; behavioral testing 24 h after the final FMT; OFT for 5 min; EPMT for 5 min; fecal collection after behavioral testing; 16S rRNA sequencing and plasma metabolite quantification. The rationale is to test whether microbiota from social anxiety disorder donors is associated with anxiety-like behavior and microbial/metabolic changes in recipient mice. | **Reported** |
| **10. Results** | For each experiment, report summary/descriptive statistics with variability and, where applicable, effect size with confidence interval. | Results 3.1-3.3; figures and tables | The revised Results include descriptive statistics and statistical test outputs for behavior, beta diversity and tryptophan metabolites, with effect sizes and confidence intervals for several behavioral and metabolite outcomes. Ensure every figure caption includes group n, statistical test, and variability measure. For microbiome comparisons, FDR/q values should be reported where applicable. | **Partially reported** |

**The Recommended Set**

| **ARRIVE item** | **Requirement from ARRIVE 2.0** | **Location in revised manuscript** | **Information reported / note for compliance** | **Status** |
| --- | --- | --- | --- | --- |
| **11. Abstract** | Provide an accurate summary of objectives, animal species, strain and sex, key methods, principal findings and conclusions. | Abstract | The abstract summarises the objective, FMT design, OFT/EPMT behavioral testing, 16S rRNA sequencing, PICRUSt2 prediction and plasma tryptophan analysis. It states that experiments were performed in antibiotic-treated mice. To fully comply, the abstract may specify the recipient animal strain and sex: two-month-old male C57BL/6 mice. | **Partially reported** |
| **12. Background** | Provide sufficient scientific background and rationale; explain how the animal species and model address the objectives and relevance to human biology where appropriate. | Introduction | The Introduction describes social anxiety disorder, limitations of current treatments, the microbiota-gut-brain axis, prior human microbiome and FMT studies, and the rationale for testing social anxiety disorder-associated microbiota in antibiotic-treated mice. It also introduces relevant mechanisms such as vagal, immune, endocrine and metabolic pathways. | **Reported** |
| **13. Objectives** | Clearly describe the research question, objectives and specific hypotheses where appropriate. | End of Introduction | The manuscript states that the primary objective was to determine whether social anxiety disorder-associated microbiota is associated with anxiety-like behaviors in recipient mice. Additional objectives were to assess gut microbial diversity, predicted functional metabolic pathways and plasma tryptophan metabolites. | **Reported** |
| **14. Ethical statement** | Provide the name of the ethical review committee that approved animal use and any relevant licence or protocol number; if approval was not sought, justify. | Sections 2.1 and 2.2 | Human-participant procedures were approved by the Medical Ethics Committee of the First Affiliated Hospital of the Air Force Medical University (KY20242376-X-2), and the trial was registered in the Chinese Clinical Trial Registry (ChiCTR2500097560). Animal procedures were approved by the Animal Care and Utilization Committee of the Air Force Medical University and conducted according to the Guidelines for the Care and Use of Experimental Animals. Add the animal protocol number if one exists. | **Partially reported** |
| **15. Housing and husbandry** | Provide housing and husbandry details, including environmental enrichment. | Section 2.2 | Mice were housed in individually ventilated cages under standard conditions (22 +/- 2 degrees C, 50 +/- 4% humidity, 12-h light/dark cycle) with free access to food and water. Cage size, bedding, number of animals per cage, diet, water type, nesting material and environmental enrichment are not fully reported and should be added if available. | **Partially reported** |
| **16. Animal care and monitoring** | Describe interventions to reduce pain, suffering and distress; report expected or unexpected adverse events; describe humane endpoints and monitoring frequency. | Section 2.2; Section 2.3 | The procedures consisted of antibiotic administration, oral gavage, non-invasive behavioral testing and sample collection. Daily monitoring, adverse events, analgesia/anesthesia if any, and humane endpoints are not fully described. Add these details only if they match the animal records. | **Partially reported** |
| **17. Interpretation / scientific implications** | Interpret results in light of objectives, hypotheses, current theory and relevant literature; discuss limitations including bias, model limitations and imprecision. | Discussion; Limitations paragraph | The Discussion interprets behavioral, microbiome and predicted functional findings in relation to the microbiota-gut-brain axis. It acknowledges key limitations: small donor cohort, antibiotic-treated rather than germ-free recipients, residual microbiota/non-bacterial microorganisms, absence of complete donor-recipient engraftment tracking, lack of social-behavior-specific tests, and reliance on PICRUSt2 inferences without multi-omics validation. | **Reported** |
| **18. Generalisability / translation** | Comment on whether and how findings generalise to other species, experimental conditions, and human biology where appropriate. | Discussion; Conclusion | The manuscript frames the findings as exploratory and relevant to the microbiota-gut-brain axis in social anxiety disorder, while limiting causal and translational claims. It states that future work should use larger donor cohorts, germ-free or gnotobiotic recipients, longitudinal sampling, strain-resolved metagenomics and social-behavior-specific paradigms before broader translation. | **Reported** |
| **19. Protocol registration** | State whether a protocol, including research question, key design features and analysis plan, was prepared before the study and where registered. | Section 2.1 | The human clinical component is registered in the Chinese Clinical Trial Registry (ChiCTR2500097560). A separate animal-experiment protocol registration is not reported. If no animal-study protocol was publicly registered before the study, state: "The animal-study protocol was approved by the institutional animal committee but was not prospectively registered in a public registry." | **Partially reported** |
| **20. Data access** | Provide a statement describing whether and where study data are available. | Data Availability Statement | The manuscript states that the data will be made available on request. If sequencing data have been deposited, add the repository name and accession number. If not, the current statement is acceptable but less transparent than repository deposition. | **Partially reported** |
| **21. Declaration of interests** | Declare financial and non-financial competing interests; list all funding sources and the role of funders. | Funding; Conflicts of Interest | No potential conflicts of interest are disclosed. Funding sources are listed: National Natural Science Foundation of China (82171512) and the Boost Plan of Xijing Hospital (XJZT25QN50 and LHJJ24YF06). Add a statement on funder role if required by the journal. | **Partially reported** |

**Suggested manuscript wording for partially reported ARRIVE items**

The following wording can be inserted into the revised manuscript where appropriate. Confirm each statement against the original laboratory records before submission.

| **Item needing confirmation** | **Suggested wording** |
| --- | --- |
| **Sample-size rationale** | The sample size was selected based on feasibility and previous fecal microbiota transplantation studies using comparable behavioral and microbiome endpoints. No formal a priori power calculation was performed; therefore, the study should be interpreted as exploratory. |
| **Inclusion/exclusion criteria** | No animals, experimental units, or behavioral/microbiome data points were excluded from the analyses. The exact n for each group is reported in the Results and figure captions. If any data were excluded, the reason and the affected outcome should be reported explicitly. |
| **Randomisation and confounder control** | After acclimatisation, recipient mice were assigned to the control or social anxiety disorder group using a random-number procedure. Behavioral testing was performed under the same environmental conditions, and the apparatus was cleaned with 70% ethanol between animals. The order of behavioral testing and sample processing should be specified if recorded. |
| **Blinding** | The experimenter was blinded to group allocation during behavioral testing and data acquisition. The blinding status during data analysis should be stated explicitly. |
| **Animal details** | Add body-weight range, substrain if available, microbiological/health status, number of animals per cage, bedding, diet, water source and environmental enrichment if these data are available. |
| **Animal care, monitoring and humane endpoints** | Animals were monitored daily for general health, body weight, food and water intake, and signs of distress. Humane endpoints included severe body-weight loss, persistent lethargy, inability to access food or water, or severe abnormal behavior. No unexpected adverse events occurred during the study. Include this statement only if it matches the actual monitoring records. |
| **Protocol registration** | The human study was registered in the Chinese Clinical Trial Registry (ChiCTR2500097560). The animal component was approved by the institutional animal committee but was not prospectively registered in a public registry. |
| **Funder role** | The funders had no role in study design, data collection, data analysis, data interpretation, manuscript preparation, or the decision to submit the manuscript for publication. Include or revise this statement according to the actual funder involvement. |

**Reference**

Percie du Sert N, Hurst V, Ahluwalia A, et al. The ARRIVE guidelines 2.0: updated guidelines for reporting animal research. PLOS Biology. 2020. doi: 10.1371/journal.pbio.3000410
